# Supplementary material for: Metabolic Profiling Analysis Reveals the Potential Contribution of Barley Sprouts against Oxidative Stress and Related Liver Cell Damage in Habitual Alcohol Drinkers
Source: Antioxidants (Basel). 2021 Mar 15;10(3):459. doi: 10.3390/antiox10030459 (PMC8000388; doi:10.3390/antiox10030459)
Supplement: Supplementary file 1 [file antioxidants-10-00459-s001.pdf]

Supplementary Table S1. Chemical fingerprints of BSE analyzed by UHPLC-LTQ-Orbitrap-MS

No.	Retention Time (min)	Tentative Metabolite	UHPLC-LTQ-Orbitrap-MS					
			$[M-H]^-$	$[M+H]^+$	M.W.	Molecular Formula	Delta ppm	MSn fragments
Phenylpropanoids								
1	2.01	Feruloylquinic acid glucoside	529.1563			C23H29O14	1.325	483.0115 437.1013 355.0922 235.0663 193.0025
2	3.79	Isoorientin 7,4′-di-O-glucoside	771.1998	773.2142	772	C33H39O21	1.01	609.3542 591.1967 447.2585 357.0911 327.0671
3	3.93	4-Feruloylquinic acid (4-FQA)	367.1044	369.1179	368	C17H19O9	0.912	321.1072 265.0999 193.2196 173.0524 134.0539
4	4.21	Isoorientin 7-O-glucoside	609.1454	611.1603	610	C27H29O16	-1.884	447.3154 357.1117 327.2857
5	4.27	Isoorientin 7-O-rhamnosylglucoside	755.2048	757.2194	756	C33H39O20	-0.128	593.1960 473.1411 447.2508 357.0753 327.1195
6	4.47	Isovitexin 7-O-glucoside	593.1504	595.1642	594	C27H29O15	-1.354	473.3727 431.4029 341.1249 311.3255
7	4.48	Isovitexin 7-O-[6″-sinapoyl]-glucoside 4′-O-glucoside	961.2615	963.2756	962	C44H49O24	-2.814	799.2409 593.3425 473.0957 367.0381
8	4.5	Isovitexin 7-O-rhamnosylglucoside	739.2081	741.2235	740	C33H39O19	-0.327	693.1055 593.1286 473.1160 431.2796 341.0977 311.1063 283.0920
9	4.56	Isoscoparin 7-O-glucoside	623.1615	625.1761	624	C28H31O16	-0.32	503.3915 461.1680 341.2623
10	4.6	Isovitexin 2″-O-glucosylarabinoside	725.2877	749.2841 (M+Na)	726	C31H49O19	1.1	679.1843 545.4150 396.0070 313.1046
11	4.73	Isoorientin 7-O-[6″-sinapoyl]-glucoside	815.2034	817.219	816	C38H39O20	0.775	461.1301 447.3273 341.0441 327.0627
12	4.84	Isoorientin 7-O-[6″-feruloyl]-glucoside	785.1932	787.2083	786	C37H37O19	0.658	461.2108 447.3177 327.2534
13	4.84	Isovitexin 7-O-[6″-sinapoyl]-glucoside	799.2088	801.224	800	C38H39O19	0.085	473.1373 431.3484 341.1075 311.2861 283.1174
14	5.03	Isovitexin 7-O-[6″-feruloyl]-glucoside	769.1986	771.2141	770	C37H37O18	-0.621	431.2868 311.2612 283.1117
15	5.32	Chrysoeriol 7-O-glucoside	461.109	463.1238	462	C22H21O11	0.619	446.3442 299.3039
16	5.32	Tricin 7-O-glucoside	491.1195	493.1344	492	C23H23O12	0.083	476.3336, 329.3528
17	6.36	Hydroxytrimethoxylflavone	327.2177	351.2144 (M+Na)	329	C18H31O5	-0.42	323.2258 209.1971 291.1740 229.1163 211.1174
Polyamines								
18	3.82	Hordatine A glucoside	757.1969 (M+FA-H)	713.3621	712	C35H49O11N8	0.769	711.5290 583.1720 549.3469 389.0996
19	4.27	Hordatine A	549.2953	551.3083	550	C28H37O4N8	0.301	507.2864 419.2284 393.4482 351.2280 237.1112
20	3.47	N-p-Coumaroylhydroxydehydroagmatine	291.147	293.1608	292	C14H19O3N4	2.529	274.1255 249.0860 171.0810 119.0206
21	4.02	p-Coumaroylagmatine	275.1518	277.1659	276	C14H19O2N4	1.493	258.1852 233.1642 215.1105 119.0752
Lipids								
22	8.15	LysoPC(18:3)	562.3140 (M+FA-H)	518.3234	517	C26H49O7NP	-1.323	502.5415 277.3071
23	8.24	PG(18:3(9Z,12Z,15Z)/0:0)	505.2559	507.2712	506	C24H42O9P	-2.519	413.2299 277.4291 227.0368 259.1875 233.1814 179.1620 191.2113 177.276

24	8.52	PI(16:0)	571.2887	573.3036	572	C25H48O12P	-0.974	409.2835 391.2624 315.0866 255.2585 241.0260
25	8.58	LysoPC(18:2)	564.3305 (M+FA-H)	520.3399	519	C26H51O7NP	-2.081	502.4280 443.2595 183.9993
26	8.69	LysoPA(18:3)	431.221	433.2348	432	C21H36O7P	3.858	395.0682 170.9535 152.9235
27	8.71	LysoPE(16:0)	452.2789	454.2926	453	C21H43O7NP	2.42	434.2776 391.2310 304.1873 255.4165
28	8.91	LysoPC(16:0)	540.3297	496.3388	495	C24H51O7NP	-4.075	478.5247 419.2274 258.0712 184.0022
29	9.12	LysoPC(18:1)	566.3453	522.3525	521	C27H53O9NP	0.168	506.5446 281.2667 224.1739
30	9.24	LysoPA(18:2)	433.2358	435.2519	434	C21H38O7P	2.016	251.1899 152.9714
31	9.9	LysoPA(16:0)	409.2366	411.06	410	C19H38O7P	0.725	227.3369 181.0214 152.9432
